# Deep Learning-Guided Identification and In Vivo Validation of Compact Cis-Regulatory Elements for the Zebrafish Habenula

**DOI:** 10.3390/genes17091079

**Published:** 2026-09-07

**Authors:** Zeran Li, Shanshan Liu, Quan Zhang, Cuizhen Zhang, Gang Peng

**Affiliations:** State Key Laboratory of Brain Function and Disorders, MOE Frontiers Center for Brain Science, Institutes of Brain Science, Fudan University, Shanghai 200032, China; 23211520018@m.fudan.edu.cn (Z.L.);

**Keywords:** cis-regulatory element, deep learning model, neural subclass, zebrafish, habenula

## Abstract

Background/Objectives: Precise genetic access to the zebrafish habenula remains limited by a scarcity of compact, sequence-defined cis-regulatory elements (CREs). Here, we integrated developmental expression mapping, deep-learning predictions on long-range sequences, and in vivo reporter assays to identify compact regulatory sequences driving habenular expression. Methods: Using a transgenic zebrafish line enriched for habenular reporter expression, we isolated GFP-positive cells from larval brains and profiled their transcriptomes via microarray. A subset of candidate genes enriched in this dataset was validated using whole-mount in situ hybridization across two developmental stages. This analysis identified genes with highly reproducible habenular expression, leading to the selection of the *gng8* and *ano2* loci for subsequent CRE characterization. We developed ZEN-former (Zebrafish EN-former), an Enformer-based sequence-to-function model trained on neuronal subclass chromatin accessibility profiles from the adult mouse brain. Results: The model demonstrated strong correlation between predicted and experimentally measured signals across held-out genomic regions. To prioritize regulatory candidates, we integrated ZEN-former predictions with available zebrafish ATAC-seq data, RepeatMasker annotations, and gene models, identifying two ~600 bp intervals at each gene locus. These selected intervals were combined to generate ~1.2 kb reporter constructs for *gng8* and *ano2* loci, which were then evaluated using Tol2 transposon mediated transgenesis assays in zebrafish. In transiently injected larvae, both constructs successfully drove reporter expression in the habenular region. Furthermore, the resulting stable transgenic lines displayed highly specific and reproducible habenular expression. Quantitative confocal analysis showed mean habenular labeling completeness values of 84.5% and 96.2% for the *gng8*- and *ano2*-derived lines, respectively. Conclusions: Together, these findings provide a proof of concept that sequence features learned from mammalian chromatin accessibility datasets can effectively guide the prioritization of functional regulatory elements across species in zebrafish. The compact regulatory constructs and stable transgenic lines generated here offer robust genetic tools for investigating habenular circuitry.

## 1. Introduction

The habenular nuclei (habenulae) are highly conserved bilaterally paired nuclei located in the dorsal diencephalon of vertebrates ranging from fish to mammals, including humans [1]. The habenula receives inputs from the limbic system and basal ganglia and projects to the dopaminergic and serotonergic centers [2]. Early work indicated that the habenula is involved in multiple behavioral processes, including anxiety-related behavior, reward evaluation, learning, and adaptive behavioral responses [3,4]. Subsequent studies have further demonstrated that the habenula plays important roles in the neural regulation of emotion, aversive processing, and affective regulation [5,6,7]. These anatomical and functional features place the habenula at a critical interface between forebrain information processing and midbrain neuro-modulation systems.

In mammals, the habenular complex can be grossly divided into medial and lateral subdivisions that differ in their afferent inputs, projection targets, molecular markers, and functional properties [8,9]. This anatomical organization is accompanied by marked cellular and molecular heterogeneity. Single-cell transcriptomic analyses have identified multiple habenular neuronal populations with distinct gene-expression programs and regulatory signatures, including patterns associated with transcription factors and markers [10,11]. However, the cis-regulatory mechanisms that establish the spatial expression patterns and cell-type-specific molecular identities of these populations remain incompletely understood [1,12].

Zebrafish have become a widely used vertebrate model in studies of molecular genetics and embryology [13,14]. This is due predominantly to its small size, high reproduction rate, and ease of genetic modification [15]. The neural circuitry and molecular expression profiles of zebrafish show substantial conservation with those of mammals. In particular, the zebrafish dorsal habenula is considered homologous to the mammalian medial habenula, whereas the ventral habenula shares major anatomical and functional features with the mammalian lateral habenula [16]. Together, the evolutionary conservation, cellular heterogeneity, optical accessibility, and genetic tractability of the zebrafish habenula make it a powerful system for investigating the molecular mechanisms underlying habenular development and function. However, such studies require genetic tools capable of selectively labeling and manipulating defined habenular cell populations.

Although promoter-fragment and enhancer-trap lines have provided partial access to zebrafish habenular populations, widely used approaches for robust habenular targeting often rely on BAC-based transgenic drivers. An approximately 200 kb BAC containing the *gng8* locus was used to generate a transgenic reporter that labels the dorsal habenular nuclei and their associated neural structures [17,18]. BAC transgenes can preserve an extended endogenous regulatory landscape and thereby reproduce complex expression patterns. However, their large size makes it difficult to identify the individual sequences responsible for cell-type-specific activity and limits their usage. Thus, compact and sequence-defined cis-regulatory elements (CREs) capable of reproducibly directing selective expression in the zebrafish habenula remain limited. Identifying such elements would provide more flexible genetic tools and enable direct investigation of the cis-regulatory logic underlying habenular expression.

Cell-type-specific chromatin accessibility maps have become an important route for translating molecular cell atlases into genetic tools. Candidate enhancers identified from scATAC-seq or snATAC-seq datasets have been incorporated into AAV vectors to provide selective genetic access to defined neuronal populations [19,20]. This strategy has since been extended to cortical interneuron subtypes, striatal cell populations, spinal motor neurons and descending motor pathways, brain endothelial cells, diverse cortical cell types, and the macaque brain [21,22,23,24,25,26]. Although chromatin-accessibility profiling can efficiently identify genomic regions enriched for potential cis-regulatory activity, accessibility alone does not establish that a sequence possesses autonomous enhancer activity or retains the expected cell-type specificity in a heterologous reporter context. In a large-scale primary in vivo screen of 682 candidate enhancers, only approximately 30% were manually classified as showing putatively on-target labeling patterns [26]. Thus, although chromatin-accessibility maps can substantially narrow the candidate space, converting accessible regions into functional cell-type-specific enhancers still requires additional prioritization and in vivo validation. Sequence-based deep-learning models offer a complementary strategy by learning regulatory features directly from DNA sequence.

The Basset model first demonstrated that convolutional neural networks could predict cell-type-resolved chromatin-accessibility signals from genomic sequence, whereas the Enformer model extended sequence-based prediction to long genomic intervals and multiple functional genomic profiles [27,28]. More recent studies have further shown that deep-learning models can learn relationships between enhancer sequence and regulatory activity and can support the identification or design of cell-type-specific CREs [29,30,31]. These advances provide a rationale for applying long-sequence models to extended genomic regions and prioritizing compact candidate CREs with predicted cell-type-specific regulatory potential. Because comprehensive, subclass-resolved transcriptomic and chromatin accessibility data are currently unavailable for zebrafish, it remains to be seen whether regulatory sequence features learned from another vertebrate, such as the mouse brain, can be applied cross-species to prioritize compact CREs in the zebrafish habenula.

In this study, we integrated developmental expression mapping in zebrafish, deep learning prediction, and in vivo reporter assays to identify compact candidate cell-type-specific CREs from genomic regions surrounding validated habenular marker loci. We first examined a candidate gene set derived from a previous enhancer-trap and microarray-based screening strategy and validated the spatial expression patterns of selected genes by whole-mount in situ hybridization. Based on the specific and reproducible habenular expression patterns, the *gng8* and *ano2* loci were selected for subsequent CRE analysis. We next utilized adult mouse brain transcriptomic and chromatin accessibility datasets resolved at the cell-subclass level to train ZEN-former, a sequence-to-function model designed to predict chromatin accessibility profiles across distinct neuronal subclasses. We applied ZEN-former to extended genomic regions surrounding the *gng8* and *ano2* loci and integrated its predictions with zebrafish ATAC-seq datasets, repetitive-sequence annotations, and gene models to prioritize compact candidate CREs. The selected sequences were evaluated in zebrafish. Evaluation included transient reporter assays, stable transgenic lines, and quantitative assessment of habenular labeling completeness. This integrated strategy identified compact regulatory sequences capable of driving reproducible habenular reporter expression and provides both new genetic tools and a general framework for converting genomic loci into experimentally validated regulatory elements.

## 2. Methods

### 2.1. Experimental Animals

This study was approved by the Ethics Committee of the Shanghai Medical College, Fudan University (approval #20150119088, 20230301130, YSA20260097) and adhered to the National Research Council’s Guide for the Care and Use of Laboratory Animals and the Fudan University Regulations on Animal Experiments.

The wild-type zebrafish AB line was obtained from the China Zebrafish Resource Center. Zebrafish were maintained in a recirculating water system under standard laboratory conditions [32]. The water temperature was kept at 28.5 °C with a 14/10 h light/dark cycle: 14 h light (08:00–22:00), 10 h dark (22:00–08:00). Embryos were raised in embryo media (5 mmol/L NaCl, 0.5 mmol/L KCl, 1.0 mmol/L CaCl_2_·2H_2_O, 0.15 mmol/L KH_2_PO_4_, 0.05 mmol/L anhydrous Na_2_HPO_4_, 1.0 mmol/L MgSO_4_·7H_2_O, and 0.83 mmol/L NaHCO_3_) at 28.5 °C before 5 days post fertilization (dpf). Phenylthiourea (PTU) was added to EM at 0.2 mM after 24 hpf to reduce pigmentation until the desired stage. Embryos were staged by days post fertilization.

### 2.2. Plasmid Construction

The enhancer constructs were built using the pminiTol2-super-Gal4FF backbone, which integrates a modified Gal4 activator (Gal4FF) and a super core basal promoter flanked by minimal Tol2 transposon elements. Candidate sequences from the *gng8* and *ano2* loci were amplified by PCR using genomic DNA extracted from AB-strain zebrafish as the template. Primer sequences are listed in Supplementary Table S1.

The amplified candidate fragments were inserted upstream of the SCP-Gal4FF cassette in the pminiTol2-super-Gal4FF backbone using the Hieff Clone^®^ Plus Multi One Step Cloning Kit (Yeasen Biotechnology (Shanghai) Co., Ltd., Shanghai, China). Purified PCR products and the linearized backbone were assembled by homologous recombination at 50 °C for 50 min. The resulting constructs were transformed into DH5α competent cells, and positive clones were identified and verified by Sanger sequencing.

The reporter construct pminiTol2-5×UAS-PM-EGFP was generated based on the PM-GFP membrane-targeting design described previously [33]. In that construct, a 10-amino-acid motif from the N-terminus of the Lyn protein, sufficient for palmitoylation and myristoylation, was fused to the N-terminus of GFP, thereby targeting GFP to the plasma membrane [34].

### 2.3. Generation of Transgenic Zebrafish Lines

Transgenic lines were generated utilizing the Tol2 transposase-mediated integration method [35,36]. Tol2 transposase mRNA was generated by in vitro transcription from a linearized mTol2 template using the mMESSAGE mMACHINE™ SP6 Transcription Kit (Thermo Fisher Scientific, Waltham, MA, USA), purified, and stored at −80 °C until use.

For microinjection, the candidate enhancer plasmid, pminiTol2-5×UAS-PM-EGFP reporter plasmid, and Tol2 transposase mRNA were mixed and injected into one-cell-stage AB zebrafish embryos. Plasmid DNA was used at 20 ng/µL each, Tol2 transposase mRNA at 80–100 ng/µL, and the injection volume was adjusted to approximately 1.5 nL per embryo.

Injected embryos were maintained under the conditions described in Section 2.1 and screened for EGFP fluorescence at 3–5 dpf. The resulting F0 founders were raised to sexual maturity and subsequently crossed with AB wild-type partners. Germline transmission was verified by screening the F1 progeny for fluorescent signals. F1 individuals were selected and cultured to establish stable transgenic lines.

### 2.4. FACS and Microarray Analysis

To identify genes enriched in habenula-associated neuronal populations, fluorescently labeled neurons were isolated from 8 dpf transgenic zebrafish larvae by fluorescence-activated cell sorting (FACS). The target population was obtained from the *etx*/*ubtor/minar1* enhancer-trap line, in which reporter expression became strongly enriched in the habenular region by 8 dpf [37]. In parallel, fluorescent cells from *lhx5*:Kaede [38,39] and S313/*nr0b1*×GFP [40] larvae were used as non-habenular reference populations. A non-fluorescent cell population was used as the control.

Larvae were anesthetized with tricaine and transferred into 15 mL ice-cold Ringer’s solution. Brain tissues were rapidly dissected and transferred into papain/DNase I dissociation solution (EBSS containing 1 mM L-cysteine, 1 mM EDTA, approximately 20 U/mL papain, and 200 U/mL DNase I). Tissues were incubated with gentle agitation for approximately 30–40 min and subsequently triturated to obtain a single-cell suspension. After allowing larger undissociated tissue fragments to settle, the supernatant containing dissociated cells was collected and centrifuged at 300× *g* for 5 min. The cell pellet was resuspended in resuspension solution (270 μL EBSS, 30 μL trypsin inhibitor solution, and 15 μL DNase I stock solution), carefully layered over the trypsin inhibitor solution, and centrifuged at approximately 70× *g* for 6 min. The cell pellet was resuspended in L-15 medium supplemented with glucose and passed through a 30 µm nylon cell strainer.

Propidium iodide (PI) was added shortly before flow cytometric sorting to facilitate exclusion of membrane-compromised cells. During FACS, the principal cell population was first selected according to Forward Scatter (FSC) and Side Scatter (SSC) characteristics. A representative FSC/SSC gating strategy and fluorescence-based sorting strategy using S313/*nr0b1*×GFP samples are shown in Figures S1 and S2. The fluorescence distribution of cells from wild-type zebrafish was used to define the background autofluorescence range and establish the fluorescence-positive sorting gate. Samples were analyzed at a flow rate of approximately 1500 events/s. Fluorescent cells (approximately 20,000 cells from each line) were collected directly into RNAqueous-Micro lysis buffer (Agilent Technologies, Santa Clara, CA, USA) for subsequent RNA purification.

Total RNA was purified using the RNAqueous-Micro RNA Isolation Kit (Ambion, Austin, TX, USA). RNA quality was assessed using an Agilent 2100 Bioanalyzer (Agilent Technologies, Santa Clara, CA, USA). Samples with an RNA integrity number (RIN) ≥ 7.0 and a 28S/18S rRNA ratio ≥ 0.7 were used for microarray analysis.

Expression profiling was performed using the Affymetrix Zebrafish Gene 1.1 ST Array Strip (Agilent Technologies, Santa Clara, CA, USA). The original dataset contained expression signals for the control, *etx/ubtor/minar1*, *lhx5*:Kaede, and S313/*nr0b1*×GFP samples and is provided as Supplementary Data S1. Genes were ranked based on their relative expression differences between the *etx* profile and the other three profiles. Candidate genes identified from this screen were subsequently examined by whole-mount in situ hybridization at 3 and 8 dpf.

### 2.5. Probe Synthesis and Whole-Mount In Situ Hybridization

Digoxigenin-labeled antisense RNA probes were synthesized using T7 or T3 RNA polymerase (Roche, Basel, Switzerland) and precipitated with LiCl. Embryos were fixed overnight in 4% paraformaldehyde in PBS at 4 °C and stored in 100% methanol. Whole-mount in situ hybridization was performed as previously described [37]. Hybridized digoxigenin-labeled probes were detected using an alkaline phosphatase-conjugated anti-digoxigenin Fab fragment (1:7500; Roche, Basel, Switzerland) and the alkaline phosphatase substrate NBT/BCIP (1:80; Roche, Basel, Switzerland). The target sequences for these probes were amplified from cDNA and cloned into the pBluescript plasmid vector. All clones were verified by Sanger sequencing. The specific primer sequences used for probe synthesis are listed in Supplementary Table S2. Whole-mount embryos were imaged using a Leica M205FA stereomicroscope equipped with a CCD camera (Leica Microsystems, Wetzlar, Germany).

### 2.6. DAPI Staining, Confocal Imaging, and Quantification of Habenular Labeling

Stable transgenic larvae at 6 dpf were fixed overnight in 4% paraformaldehyde (PFA) at 4 °C and washed three times in phosphate-buffered saline (PBS) for 15 min each. Samples were then incubated for 1 h in PBS containing 1% Triton X-100 (AMRESCO LLC, Solon, OH, USA) and DAPI (1:200), followed by three additional 15 min washes in PBS.

Fluorescence images were acquired using a Nikon A1R upright laser-scanning confocal microscope (Nikon Corporation, Tokyo, Japan) equipped with a 25× water-immersion objective.

Cell counting was performed in ImageJ (version 1.54p). The total number of DAPI-positive nuclei and the number of DAPI-positive nuclei without detectable association with membrane-localized EGFP were counted. DAPI-positive nuclei enclosed by or closely associated with membrane-localized EGFP signals were considered labeled. Habenular labeling completeness was calculated as follows:

$$Habenular\;labeling\;completeness\;=\;\frac{Total\;DAPI\;-\;unlabeled\;DAPI}{Total\;number\;of\;DAPI}\times \;100\%$$

Five independent larvae were analyzed for each stable transgenic line.

### 2.7. Dataset Acquisition and Preprocessing

The datasets used in this study were previously reported [41]. Downloaded datasets were prepared following the standard Basenji pipeline and were divided into training, validation, and test sets at ratios of 90%, 5%, and 5%, respectively. Following the configuration of Enformer, the input DNA sequence length was uniformly set to 196,608 bp to capture long-range interactions between distal regulatory elements [28].

Standard genomic sequence augmentation strategies were applied during training to expand the effective size of the training dataset, improve model generalizability, and reduce overfitting. Specifically, reverse-complement augmentation was performed to reflect the biological equivalence of the two DNA strands. Random positional shifting of the sequence windows was also applied (10 bp shifts in both directions), enabling the model to learn positionally robust sequence features.

### 2.8. Model Architecture Modification

The backbone architecture of the prediction model was derived from Enformer [28]. To adapt the model for cell-subclass-specific prediction, we modified its output module by replacing the original final projection layer of Enformer with a multi-layer perceptron (MLP) projection layer.

The final output dimension of the MLP was set to 275, corresponding to the 275 mouse whole-brain cell subclasses defined in a previous study [41]. This modification enabled the direct mapping of long-sequence representations to regulatory activity profiles across multiple cell subclasses.

### 2.9. Multi-Task Learning and Combined Loss Function

During model optimization, multi-task learning was employed to improve the robustness of the model in predicting complex transcriptional regulatory patterns. Model training used a combined loss function comprising two principal optimization tasks:

Task 1: A Poisson loss weighted by data variance was used. Because genomic sequencing count data are commonly modeled using a Poisson distribution, this loss function quantifies the difference between the predicted signal profiles and the observed values. Variance-based weighting was further introduced to balance differences in signal sparsity and overdispersion across genomic regions.

Task 2: The mean absolute error of normalized variances was used. This task was designed to constrain the accuracy with which the model captured differences in signal variability among the different cell subclasses.

The overall combined loss function was defined as follows:

$$L\;=\;\lambda \cdot L_{\mathrm{Task}1}\;+\;\left(1-\lambda \right)\cdot L_{\mathrm{Task}2}$$

### 2.10. Processing and Normalization of Genomic Tracks

To ensure comparability across different cell types and experimental datasets, all genomic coverage signals were normalized. Specifically, the signal intensity of each genomic track, including the model predictions and ATAC-seq data, was linearly scaled to the range of [0, 1] using min–max normalization:

$$X_{\mathrm{norm}}\;=\;(X\;-X_{\min})/(X_{\max}-X_{\min}\;+\;\epsilon )$$

where $\epsilon (10^{-8})$ was included to prevent division by zero.

To highlight regulatory sequences with habenula-specific activity, a percentile-based background-subtraction procedure was implemented. At each genomic position, the 90th percentile of the signal intensities across all cell-type tracks other than the target track was calculated and used as the background threshold. Signals in the target track that fell below this threshold were considered nonspecific.

## 3. Results

### 3.1. In Situ Validation of Habenula-Associated Genes

To establish an experimentally grounded set of candidate genes associated with the zebrafish habenula, we utilized a previously established Tol2-based enhancer-trap line [37]. This line, carrying a transgenic reporter insertion near the *etx*/*ubtor*/*kiaa1024/minar1* locus (current official gene symbol is *minar1*; previously used aliases include *etx/ubtor/kiaa1024*), showed reporter expression enriched in the habenular region at 8 days post-fertilization (dpf). For comparative analysis, the sorted non-fluorescent cells were used as a control. To better identify genes enriched in the habenular population, two additional transgenic lines lacking reporter expression in the habenula were included as neuronal reference populations. The cells from the *lhx5* line included neurons from the dorsal telencephalon, prethalamus, and cerebellum [38,39], whereas the S313/*nr0b1* labeled cells were predominantly hypothalamic neurons [40]. Genes were ranked based on their relative expression differences between the *etx* profile and these three comparison profiles. Several known habenula-associated genes, including *ano2*, *gng8*, and *pou4f1*, were top-ranked in this analysis (Supplementary Data S1). From this dataset, 20 candidate genes were selected by corroboration with the expression information from the Allen Mouse Brain Atlas (Table S3).

**Figure 1:**
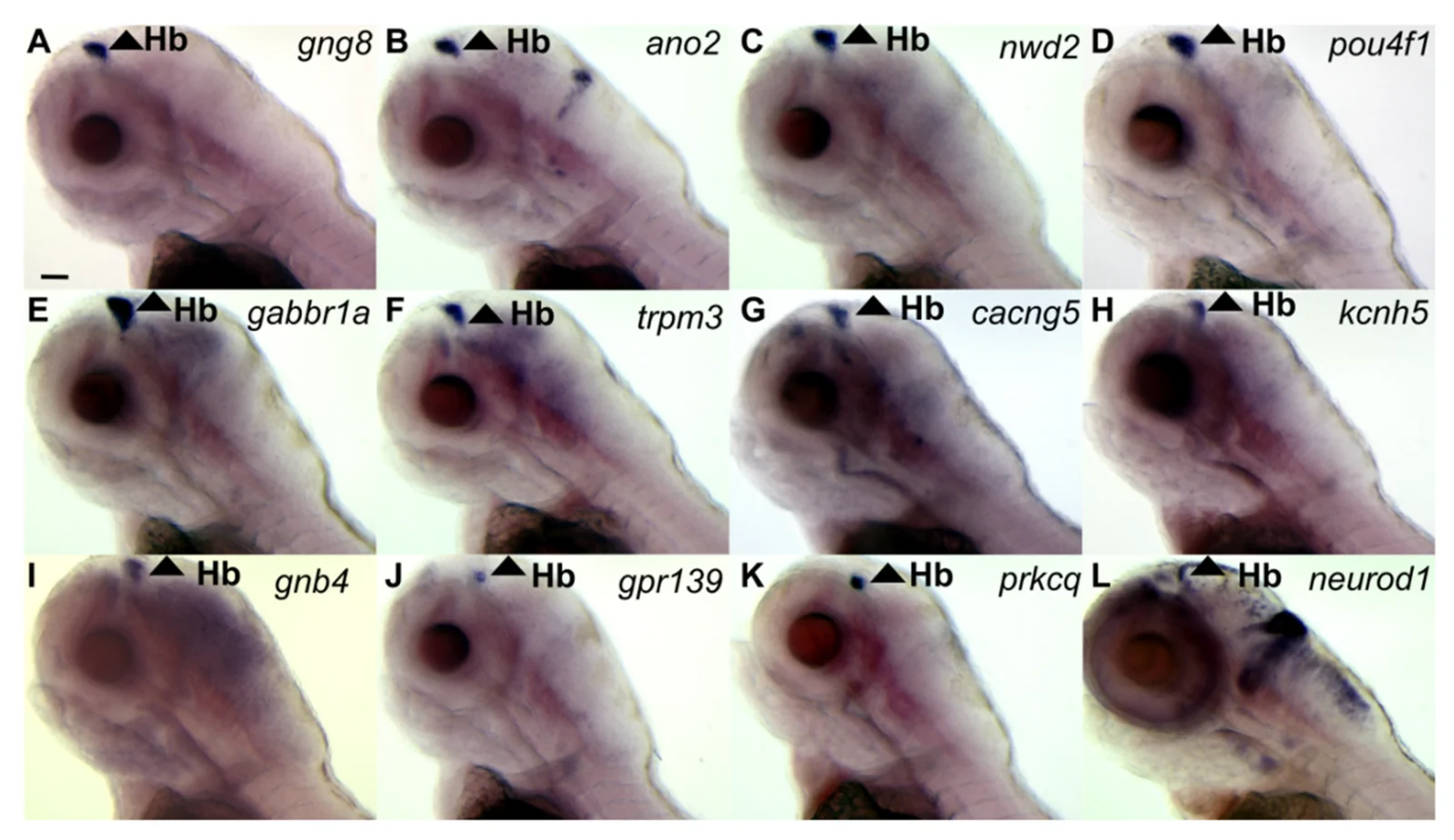
Lateral views of habenula-associated gene expression in zebrafish larvae at 3 dpf. Black arrow heads indicate the habenular region (Hb). (**A**) *gng8* showed prominent expression predominantly restricted to the habenular region, with no obvious signals detected in other parts of brain. (**B**) *ano2* expressed was concentrated in the habenula, with sporadic expressions in the tectum and mid-hindbrain boundary. (**C**–**I**) *nwd2*, *pou4f1*, *gabbr1a*, *trpm3*, *cacng5*, *kcnh5*, and *gnb4* showed enriched expression in the habenula, with lower expression levels found in the diencephalon, midbrain, and other parts of the brain. (**J**,**K**) *gpr139* and *prkcq* showed spatially restricted expression within the lateral habenular region. (**L**) As a reference, *neurod1* showed expression in the habenular regions and strong expressions in posterior and various parts of the brain. Scale bar, 100 μm.

**Figure 2:**
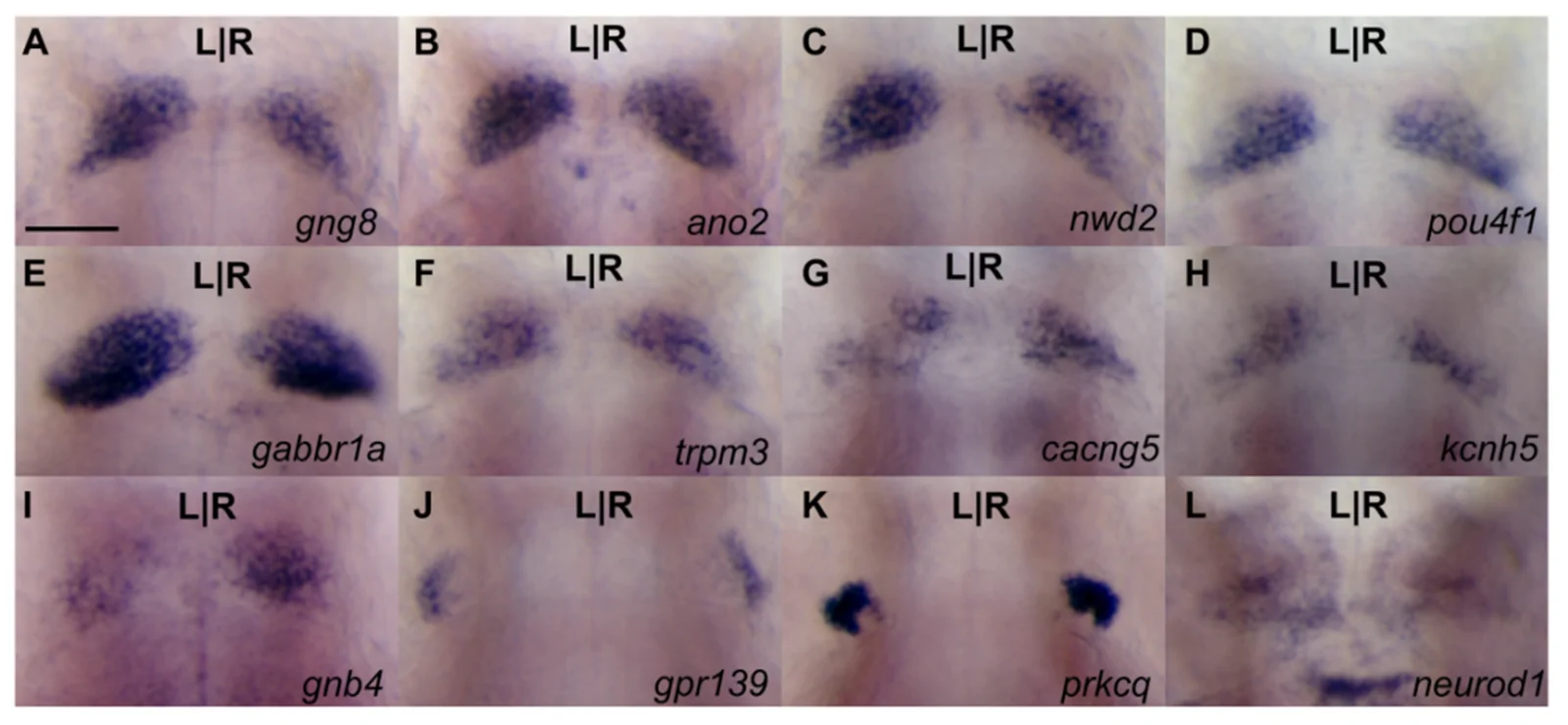
Dorsal views of habenula-associated gene expression in zebrafish larvae at 3 dpf. (**A**–**I**) *gng8*, *ano2*, *nwd2*, *pou4f1*, *gabbr1a*, *trpm3*, *cacng5*, *kcnh5*, and *gnb* showed specific and robust expression in the habenula regions. (**J**,**K**) *gpr139* and *prkcq* showed restricted expression within the lateral habenular region. (**L**) *neurod1* was expressed in the habenula and surrounding neural tissues. L and R indicate the left and right habenulae, respectively. Scale bars, 50 μm.

We assessed the spatial expression of these candidates by whole-mount in situ hybridization at 3 dpf. Twelve genes—*gpr139*, *gabbr1a*, *gng8*, *nwd2*, *gnb4*, *ano2*, *pou4f1*, *trpm3*, *cacng5*, *kcnh5*, *neurod1*, and *prkcq*—showed highly enriched expression in the habenular region (Figure 1 and Figure 2, lateral views and dorsal views, respectively).

The validated genes displayed distinct spatial-expression patterns. *gng8*, *ano2*, *nwd2*, and *gabbr1a* showed prominent bilateral expression across the habenular region. *Gnb4* exhibited an apparent left–right asymmetry, with weaker in situ signals on the left than on the right. In contrast, *gpr139* and *prkcq* were detected in more spatially restricted lateral habenular domains. We verified the specific ventral/lateral habenular localization of *gpr139* and *prkcq* transcripts within the adult zebrafish brain tissues (Supplementary Figure S3). *pou4f1*, *trpm3*, *cacng5*, and *kcnh5* were also expressed in the habenula but showed broader expression in additional neural regions. As a reference, *neurod1* transcripts were present in the habenula and also found in various brain regions. The remaining eight candidates—*olfm1a*, *cpne4a*, *vim*, *olfm2b*, *irx1a*, *cbln2a*, *fat2*, and *hs6st3*—showed broad neural expression without similarly clear habenular enrichment under the conditions examined (Supplementary Figure S4).

Because the original candidate set was derived from 8 dpf tissue, we re-examined the twelve genes at this developmental stage. Habenular expression remained detectable for all twelve genes at 8 dpf, although the spatial extent and accompanying expression in other neural regions varied among candidates (Supplementary Figure S5). Therefore, enriched habenular expression patterns were observed at both developmental stages examined.

Among the validated candidates, *gng8* and *ano2* showed strong and highly specific habenular expression at both 3 and 8 dpf. The *gng8* locus was selected because an established BAC transgenic line indicates that its broader genomic region contains regulatory information capable of driving habenular expression [17,18]. *ano2* provided an independent habenula-associated genomic context. We therefore selected the *gng8* and *ano2* loci for subsequent CRE analysis.

### 3.2. Development and Validation of ZEN-Former for Cell-Type-Specific Regulatory Profile Prediction

**Figure 3:**
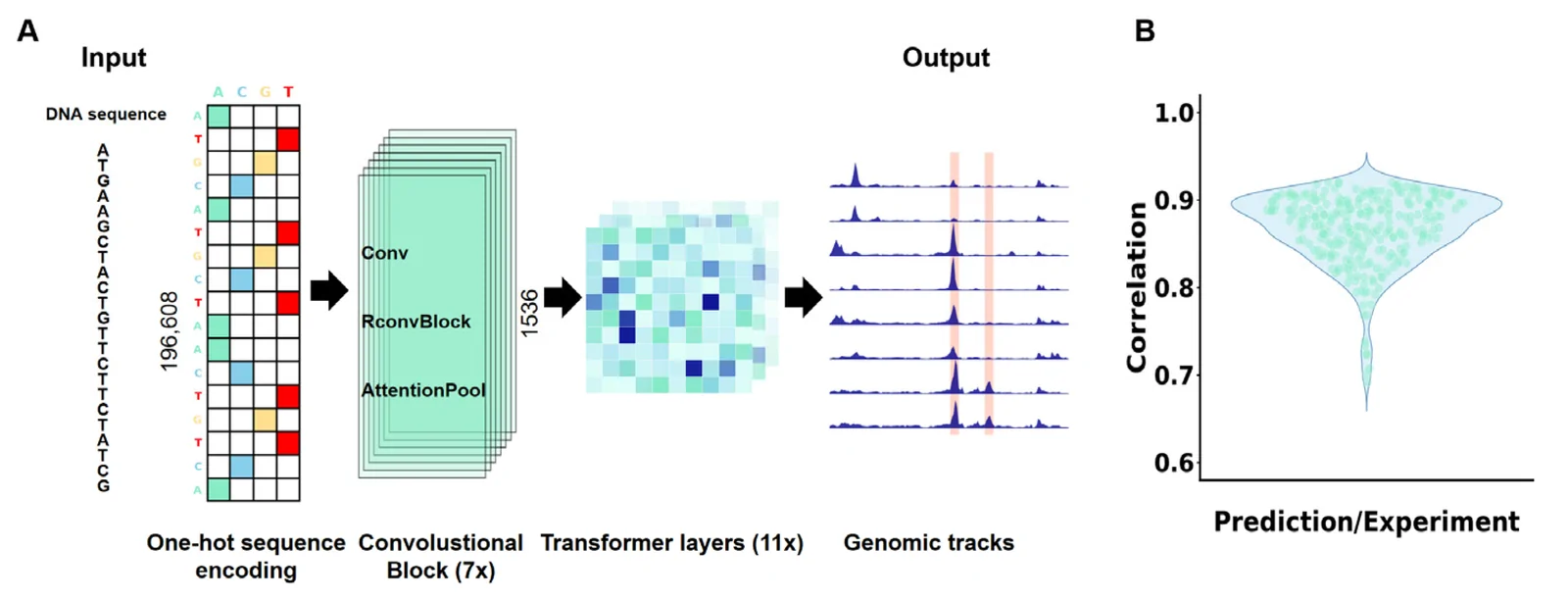
Architecture and predictive performance of ZEN-former. (**A**) Overview of the ZEN-former architecture. A 196,608 bp one-hot-encoded DNA sequence is processed through seven convolutional blocks and eleven Transformer layers to generate cell-subclass-specific regulatory profiles for 275 adult mouse brain cell subclasses in 128 bp bins. The convolutional blocks extract local sequence features and reduce the input to 1536 dimension representation, whereas the Transformer layers integrate information across the extended sequence context. (**B**) Distribution of predictive performance across the 275 cell subclasses on held-out genomic regions. Each point represents the Pearson correlation coefficient between the predicted and corresponding experimental regulatory profiles for one cell subclass. The coefficients had a mean of 0.8667, a median of 0.8766, and a range of 0.6923–0.9205.

In situ hybridization defined the endogenous spatial expression patterns of *gng8* and *ano2* but did not resolve the cis-regulatory sequences responsible for those patterns. Because relevant regulatory information may occur in intronic or distal regions across broad genomic intervals, promoter proximity alone was insufficient for systematic candidate selection. We therefore built and trained a sequence-to-function model, ZEN-former (Figure 3A), to predict chromatin-accessibility profiles across neuronal subclasses.

We took advantage of the publicly available adult mouse whole-brain single-cell transcriptomic (scRNA-seq) and single-nucleus chromatin accessibility (snATAC-seq) datasets, which provided comprehensive cell-subclass-resolved transcriptomic and chromatin accessibility data. The scRNA-seq atlas generated by Yao et al. provided the reference taxonomy and molecular annotations of brain cell types [42]. Zu et al. mapped chromatin-accessibility clusters obtained from approximately 2.3 million nuclei across 117 anatomical dissections of the adult mouse brain (approximately two months old) onto this transcriptomic taxonomy, generating aggregated snATAC-seq profiles for 275 matched brain cell subclasses [41].

These datasets were processed for model training following the standard Basenji workflow [43] and divided into training, validation, and test sets. Following the original Enformer configuration [28], the input DNA sequence length was set to 196,608 bp. This extended sequence window allows the model to integrate local sequence features with distal regulatory context over a broad genomic region. ZEN-former takes one-hot-encoded DNA sequences as input and predicts 275 cell-type-specific regulatory profiles in 128 bp bins. Similar to the Enformer model, convolutional modules extract local sequence features, whereas Transformer layers integrate information across the broader genomic context. This architecture allows the model to consider both nearby and more distant sequence features when predicting cell-type-specific regulatory activity. The resulting model contained approximately 230 million trainable parameters. Details of sequence augmentation and the training objective are provided in Section 2.7, Section 2.8, Section 2.9 and Section 2.10.

To evaluate the performance of ZEN-former, we calculated the Pearson correlation between predicted and experimental profiles for each of the 275 subclasses on genomic regions excluded from model training. The resulting coefficients had a mean of 0.8667 and a median of 0.8766. These results indicate that ZEN-former learned generalizable sequence features and reproduced cell-type-specific regulatory profiles with strong concordance between predicted and observed signals across previously unseen genomic regions.

### 3.3. ZEN-Former Identification of Candidate CREs at the gng8 and ano2 Loci

Having established the predictive performance of ZEN-former on held-out genomic regions, we next applied the model to genomic regions surrounding the zebrafish *gng8* and *ano2* loci. For each locus, we examined both the raw and class-normalized predictions across the 275 brain cell subclasses. Raw predictions represent the predicted regulatory activity of each genomic region in a given neuronal subclass. However, regions with high raw prediction signals may be active across multiple subclasses and therefore may not indicate subclass-selective regulation. To identify regulatory regions specifically associated with habenula-related subclasses, we applied class normalization across the 275 neuronal subclass predictions. This process emphasizes genomic regions showing relatively higher predicted activity in habenula-associated subclasses compared with other subclasses.

**Figure 4:**
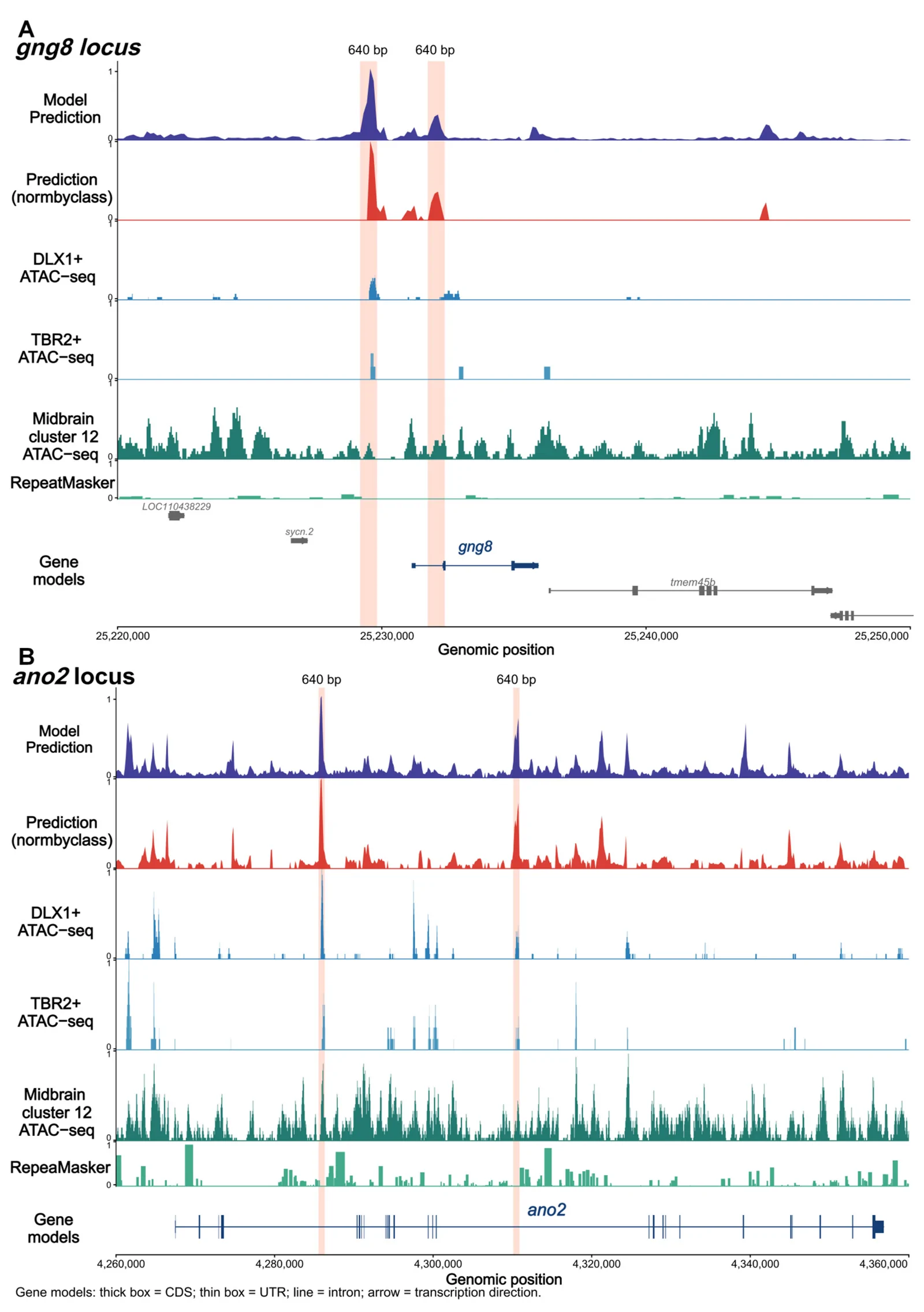
ZEN-former predictions and chromatin-accessibility profiles at the zebrafish *gng8* and *ano2* loci. (**A**,**B**) Integrated genomic views of the *gng8* (**A**) and *ano2* (**B**) loci. Tracks from top to bottom show the raw ZEN-former prediction for the habenula subclass (“Model Prediction,” dark blue), the class-normalized prediction for the habenula (“Prediction Normalized by Class,” red). In the gene-model tracks, the endogenous *gng8* and *ano2* genes are highlighted in dark blue, whereas neighboring genes are shown in gray and gene names are labelled. Thick boxes represent coding sequences, thin boxes represent untranslated regions, and connecting lines represent introns. The vertical pink-shaded regions indicate two 640 bp candidate intervals selected at each locus on the basis of predicted cell-type-specific enrichment, positional concordance with at least one independent ATAC-seq dataset, and limited repetitive-sequence content. For each locus, the two selected intervals were combined into a single candidate construct of approximately 1.2 kb for subsequent in vivo evaluation.

Predicted profiles were displayed together with three zebrafish ATAC-seq datasets derived from two published studies, RepeatMasker annotations, and gene models, all downloaded from the UCSC Genome Browser (assessable through our shared UCSC session link: https://genome.ucsc.edu/s/gpeng/danioCode7; accessed on 4 September 2026) (Figure 4A,B). The DLX1 and TBR2 tracks represent differentiating neural populations at 72 hpf [44], whereas cluster 12 represents a midbrain-associated population at 24 hpf [45]. These tracks were selected to provide complementary chromatin-accessibility references from developing zebrafish neural populations. Because none of the two studies had habenula-specific tracks, the selected tracks here were used only as supporting evidence of local chromatin accessibility rather than as evidence of habenula-specific enhancer activity.

Raw ZEN-former predictions are shown in dark blue, and class-normalized profiles are shown in red. Candidate intervals were prioritized using three criteria: (i) a prominent class-normalized prediction peak in the habenula-associated target subclasses relative to non-target subclasses; (ii) the predicted peak was required to overlap with, or show close positional concordance with, accessible chromatin detected in at least one of the independent zebrafish ATAC-seq datasets. (iii) Intervals with substantial RepeatMasker-annotated repetitive content were excluded to retain compact and uniquely mappable genomic sequences. Based on these criteria, approximately 600 bp windows centered on selected prediction peaks were defined as candidate intervals.

The *gng8* locus lies in the genomic interval covered by an established BAC-based transgenic driver that labels the zebrafish habenula [18]. At this locus, several peaks predicted by ZEN-former showed positional concordance with accessible chromatin in zebrafish differentiating neural progenitors or brain regions dataset [44,45]. We therefore selected two 600 bp intervals from the *gng8* locus; one of the selected intervals was located within an intron of *gng8*.

The *ano2* locus presented a more complex regulatory landscape (Figure 4B). The analyzed *ano2* genomic interval spanned more than 110 kb, contained nearly 30 exons, and included numerous annotated repetitive elements, making manual selection of compact regulatory sequences difficult. Despite this complexity, the class-normalized profiles highlighted two prominent localized peaks that showed predicted cell-type-specific enrichment in the habenula-associated target subclasses. We therefore identified two intronic candidate intervals at the *ano2* locus, with a combined length of 1.2 kb.

Together, these analyses prioritized two approximately 600 bp intervals at each locus. The two intervals from each locus were combined to generate one approximately 1.2 kb candidate construct, resulting in one *gng8*-derived construct and one *ano2*-derived construct. We next tested whether the predicted cell-type specificity of these constructs can drive functional enhancer activity to the zebrafish habenula in vivo.

### 3.4. In Vivo Evaluation of Candidate CREs

**Figure 5:**
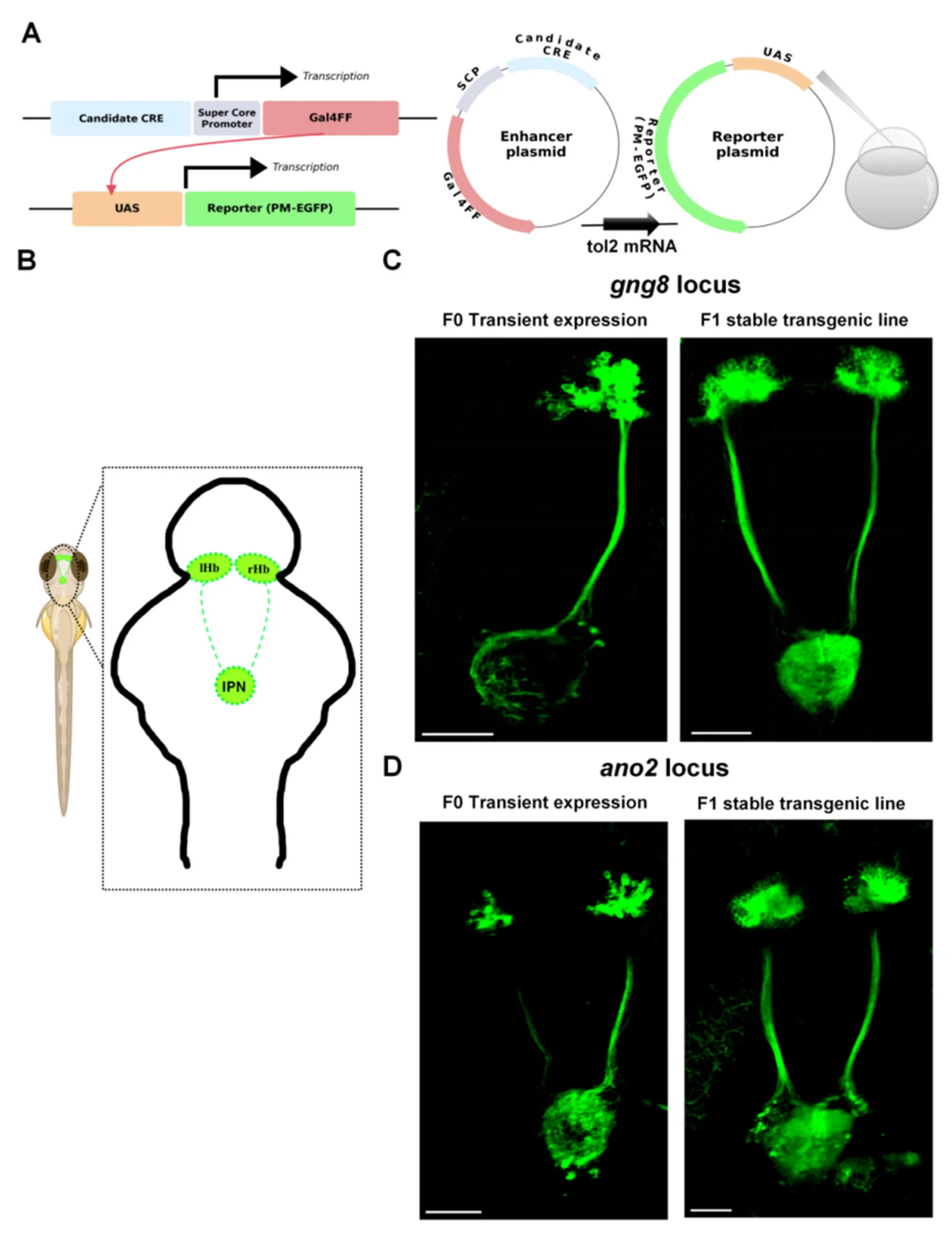
In vivo evaluation of candidate CREs in zebrafish. (**A**) Schematic of the Tol2-based Gal4/UAS reporter system and the experimental workflow. The *gng8*- or *ano2*-derived candidate CRE was cloned upstream of a super core promoter (SCP) to drive Gal4FF expression. Gal4FF subsequently binds to the upstream activating sequences (UAS) in the reporter plasmid and activates the expression of membrane-targeted EGFP (PM-EGFP). The enhancer plasmid, reporter plasmid, and Tol2 transposase mRNA were co-injected into one-cell-stage wild-type zebrafish embryos. (**B**) Schematic dorsal view of the zebrafish larval brain showing the left and right habenulae (lHb and rHb) and their descending projections to the interpeduncular nucleus (IPN). (**C**) Representative confocal images of reporter expression driven by the approximately 1.2 kb *gng8*-derived candidate sequence. The left image shows mosaic reporter expression in an F0 larva at 5 dpf, whereas the right image shows bilateral habenular expression and descending projections to the IPN in an F1 stable transgenic larva. (**D**) Representative confocal images of reporter expression driven by the approximately 1.2 kb *ano2*-derived candidate sequence. The left image shows reporter expression in an F0 larva at 5 dpf, whereas the right image shows bilateral habenular expression and descending projections to the IPN in an F1 stable transgenic larva. Scale bars in (**C**,**D**), 50 μm.

To test in vivo enhancer activity, we cloned the *gng8*- and *ano2*-derived sequences upstream of a super core promoter (SCP) and a Gal4FF cassette in a Tol2 enhancer plasmid [33]. The reporter plasmid contained UAS elements and membrane-targeted EGFP [33]. In this system, the candidate regulatory sequence and SCP drive Gal4FF expression, which subsequently activates expression of a UAS-controlled fluorescent reporter. The two plasmids were co-injected with Tol2 transposase mRNA into one-cell-stage wild-type zebrafish embryos (Figure 5A). Reporter expression was examined in the micro-injected F0 larvae at 5 dpf. The expected organization of the bilateral habenulae and their descending projections toward the interpeduncular nucleus is illustrated schematically in Figure 5B.

Both candidate CREs produced habenular reporter expression in a subset of the micro-injected F0 larvae. Among 29 F0 larvae displaying detectable EGFP expression after injection of the *gng8*-derived construct, 16 (55.2%) showed reporter expression in the habenular region. Among these, four larvae (13.8% of reporter-positive individuals) displayed expression predominantly restricted to the habenula, whereas 12 (41.4%) showed habenular expression accompanied by reporter signals in additional brain regions. The other 13 larvae (44.8%) showed mosaic reporter expression in various tissues and with little habenular labeling (Figure 5C).

Among 42 reporter-positive F0 larvae injected with the *ano2*-derived construct, 17 (40.5%) showed reporter expression in the habenular region. Three larvae (7.1%) displayed predominantly habenula-restricted expression, and 14 (33.3%) showed habenular labeling together with expression in additional brain regions. The remaining 25 larvae (59.5%) showed mosaic expressions and little habenular reporter expression (Figure 5D).

As expected for F0 Tol2 reporter assays, the distribution of EGFP expression varied among individual larvae. Nevertheless, the detection of habenular reporter activity from both constructs supports the ability of ZEN-former to identify compact genomic sequences with cell-type-specific regulatory potential in vivo.

To overcome the mosaic expression and to determine whether the reporter patterns could be stably transmitted through the germline, EGFP-positive F0 larvae were raised to adulthood and outcrossed to wild-type fish, and their F1 progeny were screened for EGFP fluorescence. Germline transmission was obtained for both the *gng8*- and *ano2*-derived constructs, resulting in stable F1 transgenic lines. Compared with the heterogeneous expression patterns observed in F0 larvae, reporter expression in F1 progeny was highly specific and uniform among individuals. Among 42 EGFP-positive F1 larvae derived from the *gng8* founders, all 42 displayed habenula-restricted reporter expressions. Similarly, all 24 EGFP-positive F1 larvae derived from *ano2* founders showed specific and uniform reporter expression in the habenular region (Figure 5C,D).

Whole-larva imaging of the established stable lines further revealed prominent habenular reporter expression together with additional expression domains (Figure S6). In the *gng8*-derived line, additional reporter expression was observed in the olfactory region, consistent with the reported endogenous expression of *gng8* in the zebrafish olfactory system. In the *ano2*-derived line, additional reporter expression was detected in a region between the optic tectum and cerebellum, consistent with the endogenous *ano2* expression pattern observed in Figure 1B.

**Figure 6:**
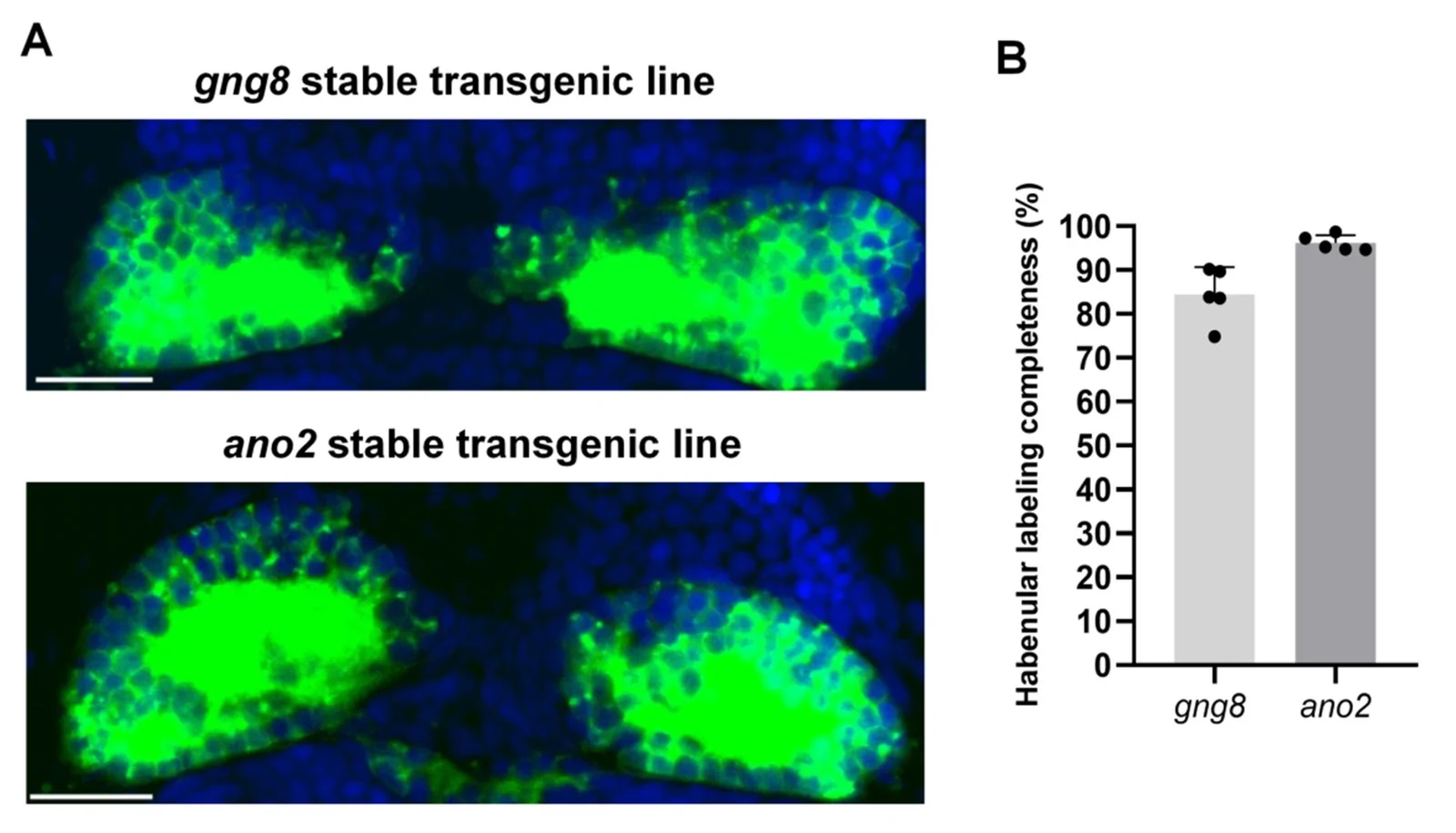
Quantitative assessment of habenular labeling completeness in stable transgenic zebrafish driven by *gng8*- and *ano2*-derived candidate CREs. (**A**) Representative confocal images of reporter expression in the habenular region in 6 dpf stable transgenic larvae driven by *gng8*- or *ano2*-derived candidate sequence. DAPI-positive nuclei are shown in blue, and membrane-localized EGFP is shown in green. Scale bars, 10 μm. (**B**) Quantification of habenular labeling completeness in the stable transgenic lines. Labeling completeness was calculated as the percentage of DAPI-positive nuclei associated with membrane EGFP labeling among all DAPI-positive nuclei within the anatomically defined habenular region. The labeling completeness was 84.5 ± 6.2% for the *gng8*-derived line and 96.2 ± 1.8% for the *ano2*-derived line. Each dot represents one independent larva. Bars indicate the mean, and error bars indicate SD; *n* = 5 larvae per transgenic line.

To further quantify the extent of habenular labeling in the stable transgenic lines, 6 dpf F1 larvae were examined by confocal microscopy following DAPI counterstaining (Figure 6A). Membrane-localized EGFP was associated with a large proportion of DAPI-positive nuclei within the anatomically defined habenular region in both lines. Habenular labeling completeness was calculated as the percentage of DAPI-positive nuclei associated with membrane EGFP labeling among all DAPI-positive nuclei within the habenular region. The mean labeling completeness was 84.5 ± 6.2% for the *gng8*-derived line and 96.2 ± 1.8% for the *ano2*-derived line (mean ± SD, *n* = 5 independent larvae per line; Figure 6B). These results demonstrate robust and reproducible labeling of the habenular region in both stable transgenic lines.

Together, the in vivo F0 and F1 reporter assays provide experimental evidence that ZEN-former can identify compact genomic sequences capable of supporting reproducible habenular reporter expression in the zebrafish habenula. By converting habenula-associated genomic loci into experimentally validated regulatory constructs approximately 1.2 kb in length, this study provides compact genetic tools for accessing the zebrafish habenular circuit and supports the broader application of sequence-based models to cell-type-specific CRE discovery.

## 4. Discussion

In this study, we established a workflow that links developmental expression mapping, deep learning regulatory-profile prediction, and in vivo reporter validation to identify compact candidate cell-type-specific CREs associated with the zebrafish habenula. Spatial screening identified a panel of habenula-associated genes and supported the selection of the *gng8* and *ano2* loci as representative and complementary regulatory contexts. ZEN-former-guided candidate intervals were analyzed from both loci. The resulting constructs drove habenular reporter expression in transient assays and stable transgenic lines, with quantitative confocal analysis further demonstrating broad labeling of the habenular region.

The expression pattern analyses provided an experimentally grounded bridge between the initial enhancer-trap screen and subsequent CRE prioritization. Candidate genes derived from the enhancer-trap and microarray screen were evaluated in parallel by whole-mount in situ hybridization at 3 and 8 dpf, revealing a range of bilateral, asymmetric, spatially restricted expression patterns in the habenula. By examining a candidate panel under matched experimental conditions, this study generated a spatial reference for habenula-associated gene expression during larval development. Within this panel, *gng8* and *ano2* displayed highly specific and robust habenular expression at both developmental stages and therefore provided two complementary genomic contexts for subsequent ZEN-former-guided CRE prioritization.

Conventional CRE discovery strategies commonly rely on promoter proximity, cross-species sequence conservation, chromatin-accessibility profiling, or empirical testing of multiple genomic fragments. Promoter-centered searches may miss distal regulatory elements, including long-range enhancers located far from their target genes [46]. Conservation-based analyses may overlook functional enhancers with weak primary-sequence conservation [47]. Moreover, chromatin accessibility can identify candidate regulatory regions, but it does not by itself demonstrate autonomous enhancer activity or determine cell-type-specific expression in a reporter context [48]. Functional validation is therefore required to assess candidate activity and specificity [19]. Thus we established and employed the ZEN-former model to predict CREs in habenula subclasses. These predictions were further integrated with zebrafish ATAC-seq signals, RepeatMasker annotations, and gene models to provide additional support and assess the feasibility of candidate sequences for experimental testing.

The *gng8* and *ano2* loci illustrate two complementary applications of this strategy. At the *gng8* locus, a BAC-based transgenic driver [17] and a 3060 bp promoter construct had previously supported habenular reporter expression [49]. Interestingly, this 3060 bp region contains both 600 bp intervals in our approximately 1.2 kb construct, providing independent contextual support for the selected sequences and suggesting that ZEN-former identified a more compact candidate sequence within a previously functional regulatory region. By contrast, the *ano2* locus presented a broader and more repetitive genomic landscape. Here, the same strategy focused experimental testing on two localized intronic intervals that supported habenular reporter expression. Together, *gng8* represents refinement within a previously functional regulatory domain, whereas *ano2* represents candidate within a structurally complex and less characterized locus.

The heterogeneous reporter patterns observed in F0 larvae are consistent with the mosaic and variable expression commonly encountered in transient Tol2-based reporter assays [50,51]. Germline transmission and the highly reproducible F1 expression patterns provide stronger evidence that the regulatory activity of the selected constructs can be stably inherited and support the use of the resulting lines as genetic access points for studying the zebrafish habenula. Quantitative confocal analysis further showed that the two stable lines labeled a large proportion of DAPI-positive nuclei within the habenular region. The mean labeling completeness values were 84.5% for the *gng8* line and 96.2% for the *ano2* line. These findings extend our qualitative F1 observations by demonstrating broad anatomical coverage of the habenula in these stable transgenic lines. Notably, subtle differences emerged between the two lines. The *ano2* line showed a small number of GFP-positive cells distributed near the habenular regions, whereas the *gng8* line lacked GFP-positive cells outside the habenula. These variations closely mirrored endogenous expression patterns: *ano2* transcripts were concentrated in the habenula with scattered expression in the tectum and other brain regions, whereas *gng8* expression was predominantly restricted to the habenula. Overall, the present work establishes habenula-selective anatomical expression, it does not resolve specificity among molecularly defined habenular cell types, which remains a direction for future work.

The application of ZEN-former, which was trained on adult mouse brain data, to zebrafish genomic loci extends the model beyond its original training species and cellular context. Previous computational and functional studies have demonstrated that regulatory information learned or identified in one species can, in some contexts, assist in regulatory prediction or cell subclass selective targeting in another species [20,52]. The present study suggests that sequence features learned from mammalian brain data can provide useful information for recognizing regulatory candidates in zebrafish. However, ZEN-former outputs should be interpreted as hypothesis generating predictions of cell type specific regulatory potential rather than as direct measurements of zebrafish chromatin accessibility or cellular identity. This limitation arises from the lack of zebrafish comprehensive neural subtype resolved chromatin accessibility datasets, which will be important for future calibration and refinement of ZEN-former’s cross-species predictive performance.

Several directions will be important for extending the present proof-of-concept study. First, the current in vivo validation was limited to two constructs derived from the *gng8* and *ano2* loci, and the validated loci were selected based on prior habenular expression information. Therefore, the present design does not directly benchmark ZEN-former against promoter cloning strategies or establish its ability to identify CREs without prior knowledge of target cell-type expression. Future studies should evaluate larger and more diverse candidate sets across additional loci, including candidates with high, intermediate, and low prediction scores. Moreover, using unbiased genome-wide candidate selection, non-predicted genomic controls, and systematic comparisons with conventional approaches will be necessary to further evaluate the predictive specificity and broader applicability of ZEN-former. Massively parallel reporter assays could also complement in vivo testing by enabling larger candidate libraries to be evaluated in parallel [53]. Another limitation is that reporter-positive cells were identified as habenular based on anatomical location, without direct co-localization with endogenous *gng8* or *ano2* transcripts or an independent habenular marker. In zebrafish, the habenula is anatomically situated in the dorsal-most thalamic region and distinctly separated from adjacent brain areas, so the confidence in these reporters correctly identifying the habenula remains high. Nevertheless, the DAPI-based analysis reflects reporter coverage within the anatomically defined habenular region rather than cellular-level correspondence with endogenous gene expression. Future double-labeling experiments will be necessary to assess this correspondence directly.

Second, each approximately 1.2 kb construct combined two 600 bp intervals, leaving the individual contribution of each interval and any potential cooperative effects unresolved. Testing the individual intervals and their different combinations will help define the minimal sequence requirements for habenula-associated regulatory activity. In silico saturation mutagenesis and motif analysis could then be used to identify candidate nucleotides and transcription factor binding sites associated with the predicted cell type specific activity [28]. Such experiments would distinguish the sequence determinants of CRE activity from the broader roles of the corresponding transcription factors in habenular development and function.

Third, functional testing in mammalian systems will be important for determining whether the cross-species utility of ZEN-former extends beyond candidate prioritization in zebrafish. Regulatory sequences could be incorporated into AAV reporter vectors and evaluated in mice using local brain delivery or an appropriately validated systemic delivery strategy [19].

## 5. Conclusions

In conclusion, this study integrated developmental expression mapping, ZEN-former-guided regulatory-profile prediction, and zebrafish transgenic assays to prioritize compact candidate CREs from the *gng8* and *ano2* loci. Both approximately 1.2 kb constructs supported highly specific and robust habenula reporter expression and germline transmission. The resulting compact CREs and stable transgenic lines provide new genetic entry points for labeling and investigating the zebrafish habenula. More broadly, these findings provide proof of concept for using a long-sequence deep-learning model for cell-type-specific regulatory prediction to guide the conversion of extended genomic regions into compact, experimentally validated regulatory tools.

## Data Availability

The data presented in this study are included in the article and Supplementary Materials. Additional raw imaging and quantitative data are available from the corresponding author upon reasonable request. Publicly available datasets analyzed in this study are cited in the Materials and Methods.

## Supplementary Materials

The following supporting information can be downloaded at https://www.mdpi.com/article/10.3390/genes17091079/s1, Figure S1. FSC/SSC-based gating strategy for zebrafish brain cell suspension preparation; Figure S2. Establishment of fluorescence-positive gates using wild-type and GFP-expressing zebrafish samples; Figure S3: Expression of gpr139 and prkcq in the habenula of adult zebrafish; Figure S4: Lateral views of candidate gene expression in zebrafish larvae at 3 dpf; Figure S5: Dorsal views of habenula-associated gene expression in zebrafish larvae at 8 dpf; Figure S6. Whole-larva expression patterns of 5 dpf F5 gng8 and ano2 CRE reporter lines. Table S1: Primer sequences used for amplification and assembly of the gng8- and ano2-derived candidate CRE constructs; Table S2: Primer sequences used for antisense RNA in situ probe synthesis; Table S3: Candidate genes selected from the microarray-derived gene expression dataset. Supplementary Data S1: the original dataset contained expression signals for the control, *etx/ubtor/minar1*, *lhx5*:Kaede, and S313/*nr0b1*×GFP samples.
