# Supplementary material for: Deep Learning-Guided Identification and In Vivo Validation of Compact Cis-Regulatory Elements for the Zebrafish Habenula
Source: Genes (Basel). 2026 Sep 7;17(9):1079. doi: 10.3390/genes17091079 (PMC13606746; doi:10.3390/genes17091079)
Supplement: Supplementary file 1 [file genes-17-01079-s001.zip › genes-4497627-supplementary.docx]

**Figure S1. FSC/SSC-based gating strategy for zebrafish brain cell suspension preparation. Representative FSC/SSC plot showing the identification of the main cell population from dissociated zebrafish larval brain samples. The FSC-H/SSC-H gate was used to exclude debris and large aggregates and to define the cell population subjected to subsequent sorting.**

**Figure S2. Establishment of fluorescence-positive gates using wild-type and GFP-expressing zebrafish samples.** (A) Representative FL-GFP distribution of wild-type zebrafish brain cells used to determine background autofluorescence levels. (B) Representative FL-GFP distribution of dissociated S313/*nr0b1*×GFP brain cells showing the fluorescence-positive sorting gate. The gate was defined based on comparison with wild-type autofluorescence controls.

**Figure S3. Expression of gpr139 and prkcq in the habenula of adult zebrafish.** Coronal sections of brains from 4-month-old wild-type AB zebrafish are shown. Black boxes indicate the habenular region (Hb). (A,B) Overview of gpr139 and prkcq expression in the adult zebrafish brain, showing prominent expression in the habenula. (C) Enlarged view of the habenular region in (A), showing that the gpr139 in situ hybridization signal was predominantly concentrated in the ventral habenula. (D) Enlarged view of the habenular region in (B), showing that the prkcq in situ hybridization signal was predominantly concentrated in the ventral habenula.

Dashed outlines indicate the approximate boundaries of the habenular region. Scale bars: 100 μm in (A, B); 50 μm in (C, D).

**Figure S4. Lateral views of candidate genes showing neural expression in zebrafish larvae at 3 dpf.** (A) olfm1a, (B) hs6st3, (C) cpne4a, and (F) cbln2a showed expression in the olfactory bulb and diencephalon. olfm1a and cpne4a were broadly expressed throughout the diencephalon, whereas hs6st3 and cbln2a showed stronger expression in ventral diencephalic regions. cbln2a also showed an additional discrete signal in another neural region. (E, G) olfm2b and fat2 were mainly expressed in ventral diencephalic regions. (D) irx1a showed broad expression throughout the diencephalon. (H) vim showed scattered expression throughout the head. Scale bar, 100 μm.

**Figure S5. Dorsal views of habenula-associated gene expression in zebrafish larvae at 8 dpf.** L and R indicate the left and right habenulae, respectively. (A–H, J–L) gng8, ano2, nwd2, pou4f1, gabbr1a, trpm3, cacng5, kcnh5, prkcq, gpr139, and neurod1 showed no obvious left-right bias in habenular expression. (I) gnb4 showed stronger expression in the right habenula. (J, K) prkcq and gpr139 showed spatially restricted expression in the lateral regions of the habenulae. Scale bar, 50 μm.

**Figure S6. Whole-larva expression patterns of 5 dpf F5 *gng8* and *ano2* CRE reporter lines. Reporter-positive habenular neurons (Hb) and their projections to the interpeduncular nucleus (IPN) are indicated by arrows. The IPN is a major projection field of the habenula.** (A) Lateral view of an F5 gng8 reporter larva showing prominent reporter expression in the habenular region and additional expression in the olfactory bulb (ob). The olfactory expression domain is consistent with the reported endogenous expression pattern of gng8. (B) Lateral view of an F5 ano2 reporter larva showing prominent habenular expression together with additional reporter expression in a brain region between the optic tectum and cerebellum, consistent with the endogenous ano2 expression pattern observed in this study. The notochord is also labeled in this transgenic line. (C,D) Enlarged lateral views of the anterior brain regions of the *gng8* and *ano2* reporter larvae, respectively. Additional non-target expression domains in the brain are indicated by asterisks. Anterior is to the left and dorsal is up. Scale bars, 500 μm (A,B) and 100 μm (C,D).

**Table S1. Primer sequences used for amplification and assembly of the *gng8*- and *ano2*-derived candidate CRE constructs.** Lowercase letters indicate homologous overlap sequences introduced for seamless assembly, whereas uppercase letters indicate template-specific primer-binding sequences used for PCR amplification.

| **Gene** | **Primer** | **Sequences ( 5' to 3')** |
| --- | --- | --- |
| *gng8* | *gng8*-1F | GCCCAGTTTAATTTaactagaagtcaagttattgtttgc |
|  | *gng8*-1R | caaaaaaatcctgTATTTACAAAGCAGATTAACATGCCC |
|  | *gng8*-2F | CTGCTTTGTAAATAacaggatttttttgagctgagtttc |
|  | *gng8*-2R | gtacgggatctatttCTGCTCCACTGTTTTTCGTG |
|  | p-mTol2-*gng8*-up | acagtggagcagAAATAGATCCCGTACTTATATAAGGGG |
|  | p-mTol2-*gng8*-dw | cttgacttctagttAAATTAAACTGGGCATCAGCG |
| *ano2* | *ano2*-1-F | ccagttttaATTAATGCAATCCAATTTATATTGTTC |
|  | *ano2*-1-R | gtttgtttgtttgtGATCTCTTTTTGCGAGTTGTAG |
|  | *ano2*-2-F | caaaaagagatcACAAACAAACAAACAAACAAAAAAAAACTTG |
|  | *ano2*-2-R | cgggatctatttCAGATTGCTATTTGACTGTGAGTTC |
|  | p-mTol2-*ano2*-up | aatagcaatctgAAATAGATCCCGTACTTATATAAGGGG |
|  | p-mTol2-*ano2*-dw | gattgcattaatAAATTAAACTGGGCATCAGCG |

**Table S2. Primer sequences used for antisense RNA in situ probe synthesis.**

| **Gene** | **Forward primer (5′–3′)** | **Reverse primer (5′–3′)** |
| --- | --- | --- |
| *ano2* | GTATTCTCCTCTCACCTGTTCAG | GATTTAACTTCCGCCACACTTC |
| *cacng5* | GCTGGATTACACTCACATCAC | TAAGGAGGGCAGGGTAACTG |
| *cbln2a* | GAGAGGAGAGAGGAAGATTGAG | CGAGTAACAGCGTTCTGCTTC |
| *cpne4a* | ACAACGGCATGAGAGAGGCG | TTCACGGGGTCGCTCAAACTCA |
| *dachb* | TGTCCTGGGAGAGAGAGAAAC | TCGTGAGGGCTTTAGACTGAAG |
| *gabbr1a* | AAACTGGGCAAATGGGACTCACC | CGTACCAGGACAGGTTGCCTT |
| *gnb4* | ATCAGTATGGGTGTGGTAATCG | AGCGTAGGCACAAGTCATCAC |
| *gng8* | CTGATGGCTTACTGTGAATCTC | GCCTTGGAGCTTACTGACTTG |
| *gpr139* | GGCAGAAATCCTCCTACAACTAC | GTAAACCATTGCCTACATCGCT |
| *hs6st3* | GGTCAGGCTTGTCATTTCAGG | ACCAGTCGTCACCGTTGTAG |
| *ica1* | ACCCGCAAACTAAAGGATTACC | TGACGCTCCAGAGACTCATTC |
| *irx1a* | TGGGCTACCCGCAGTATTTAAGT | GCTGTCCCGCCAAAAGTTGA |
| *kcnh5* | TGTCCTGGGAGAGAGAGAAAC | TCGTGAGGGCTTTAGACTGAAG |
| *neurod1* | CACTGGAGCCGTTCTTTGAC | GCCTATTCCCACCTGTGTTTG |
| *nwd2* | CATGAGACTGGCAGATGGTAAG | GAAACTCGGGATGACTTCAGAC |
| *olfm1a* | TCTCATCTTCACCTCATTCTGC | GTCTGATGGCAACTCATCTAGGA |
| *olfm2b* | GAGAAGATCATCAGCGTGTTGTAC | GGTGTGTGTGTGTGTATGTAGTG |
| *pou4f1* | CTGAGCACAAGTACACAACTCT | GGCTATTTCTCGTCATTCATCG |
| *prkcq* | CCTGAACAAACAGGGATACCAG | CCAAATGACCACCAGTCTACAG |
| *vim* | ACCCGCAAACTAAAGGATTACC | TGACGCTCCAGAGACTCATTC |

**Table S3. Candidate genes selected from the microarray-derived gene expression dataset.** Most corresponding mouse orthologs show expression in the habenula according to the Allen Mouse Brain Atlas. Expression patterns can be accessed at <https://mouse.brain-map.org/>.

| **Official symbol** | **Zebrafish gene symbol** | **Full name** | **Mouse habenular expression (Allen Brain Atlas)** |
| --- | --- | --- | --- |
| ANO2 | *ano2* | anoctamin 2, calcium-activated chloride channel | Yes |
| CACNG5 | *CACNG5(LOC100331116)* | calcium channel, voltage-dependent, gamma subunit 5 | Yes |
| CBLN2 | *cbln2a* | cerebellin 2 precursor | Yes |
| CPNE4 | *cpne4a* | copine IV | Yes |
| FAT2 | *fat2* | FAT atypical cadherin 2 | No |
| GABBR1 | *gabbr1a* | gamma-aminobutyric acid (GABA) B receptor | Yes |
| GNB4 | *GNB4(LOC100332205)* | guanine nucleotide-binding protein (G protein), beta polypeptide 4 | Yes |
| GNG8 | *gng8* | guanine nucleotide-binding protein (G protein), gamma 8 | No |
| GPR139 | *GPR139(LOC798483)* | G protein-coupled receptor 139 | Yes |
| HS6ST3 | *hs6st3* | heparan sulfate 6-O-sulfotransferase 3 | Yes |
| IRX1 | *irx1a* | iroquois homeobox 1 | Yes |
| KCNH5 | *KCNH5(LOC100331767)* | potassium voltage-gated channel, subfamily H, member 5 | Yes |
| NEUROD1 | *neurod1* | neuronal differentiation 1 | Yes |
| NWD2 | *LOC562320* | NACHT and WD repeat domain-containing protein 2 | Yes |
| OLFM1 | *olfm1a* | olfactomedin 1 | Yes |
| OLFM2 | *OLFM2(zgc:85900)* | olfactomedin 2 | Yes |
| POU4F1 | *pou4f1* | POU class 4 homeobox 1 | Yes |
| PRKCQ | *prkcq* | protein kinase C, theta | Yes |
| *TRPM3* | *trpm3* | Transient Receptor Potential Cation Channel Subfamily M Member 3 | Yes |
| VIM | *vim* | vimentin | No |
